# A Memorial to Dr. Joseph O'Dwyer

**Published:** 1899-06

**Authors:** 


### A Memorial to Dr. Joseph O'Dwyer.

A representative committee has been formed for the purpose of doing honour to the memory of Dr. Joseph O'Dwyer. Dr. Geo. F. Shrady has been elected permanent Chairman, and Dr. Alfred Meyer permanent Secretary, and the following Committee on Scope and Plan was appointed: Dr. Dillon Brown, Chairman, and Drs. Robert Abbe, R. G. Freeman, L. Emmet Holt, and Louis Fischer. The memorial will probably take an educational form.

